# Role of Next-Generation Sequencing in the Treatment of Metastatic Apocrine Carcinoma: A Rare Entity

**DOI:** 10.7759/cureus.76115

**Published:** 2024-12-21

**Authors:** Ammara Yasmeen, Midhat Waheed, Muhammad Awais Majeed, Naqib Ullah, Muhammad Mudasir, Sameen Bin Naeem

**Affiliations:** 1 Oncology, Shaukat Khanum Memorial Cancer Hospital and Research Centre, Lahore, PAK; 2 Oncology, Shaukat Khanum Memorial Cancer Hospital and Research Center, Lahore, PAK; 3 Gastroenterology, Shaukat Khanum Hospital and Research Centre, Lahore, PAK

**Keywords:** apocrine, chemotherapy, next generation sequencing (ngs), radiotherapy, somatic mutations

## Abstract

Primary apocrine adenocarcinoma, a rare malignancy, is an aggressive tumor rarely reported. It has diagnostic and treatment challenges as it is difficult to distinguish it from metastases due to breast carcinoma. Currently, no data are available for the use of next-generation sequencing to identify the possibility of targeted therapies for metastatic disease. Only a few cases are reported, hence, no consensus has been developed for treatment guidelines, and a prognosis has not been established. We present here a case of this rare tumor, its treatment course, and the role of next-generation sequencing for the use of targeted therapy.

## Introduction

Primary apocrine adenocarcinoma is a rare malignancy that accounts for 0.005-0.017 per 100,000 patients per year [1]. Only a few case reports are published in the literature with no consensus guidelines for its diagnosis and management, with most cases from the axilla [2-13]. It usually occurs in the axillae and the anogenital region, which have significant apocrine gland densities. There are case reports in the literature with apocrine adenocarcinoma of different parts of the body, including extremities, scalp, eyelids, ear canal, nipples, and chest too [4, 8, 10, 14-16]. The patient can be asymptomatic or can present with a painful nodule or cystic mass, erythematous to violaceous in color, with overlying granulation tissue and slow-growing, sometimes having purulent discharge from the lesion [6, 9, 11]. Wide local excision with clear margins is the standard treatment approach for non-metastatic localized apocrine adenocarcinoma; however, there is no consensus on the management of metastatic disease. However, some case reports have established the role of chemotherapeutic agents (anthracyclines, taxanes, and platinum compounds), anti-HER-2-directed therapies, and endocrine therapies [17]. Currently, no data are describing the role of next-generation sequencing in metastatic apocrine cancers for the targeted therapies that may be related to tumor progression or drug resistance, as in recurrent/metastatic breast cancers proven to have additional genetic changes compared with the primary tumor [18]. We present the case of a patient who presented with a left axillary lump, diagnosed with apocrine adenocarcinoma, and treated as mammary carcinoma.

## Case presentation

**Figure 1:**
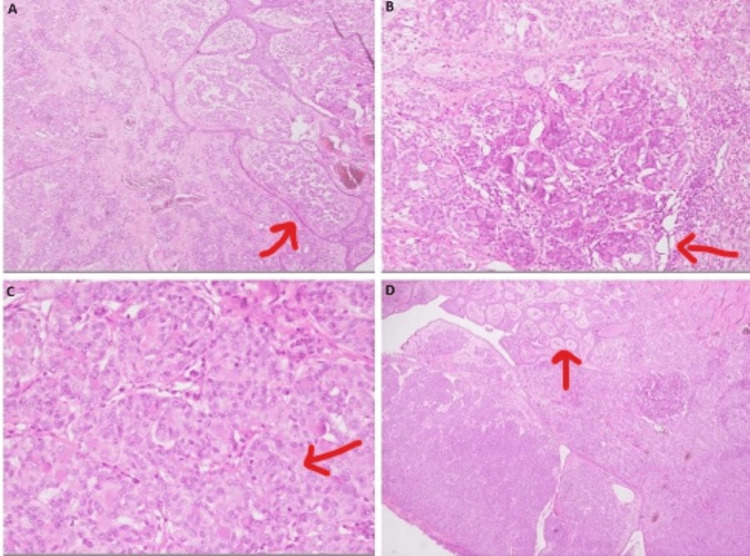
Histopathological images of the tumor A. Sections reveal a malignant neoplasm composed of clusters, nests, and infiltrative cords of moderately atypical tumor cells. B. There are areas with gland formation. C. Both intracellular and extracellular mucin is identified. D. Tumor cells have eosinophilic cytoplasm, irregular nuclei, and vesicular chromatin.

**Figure 2:**
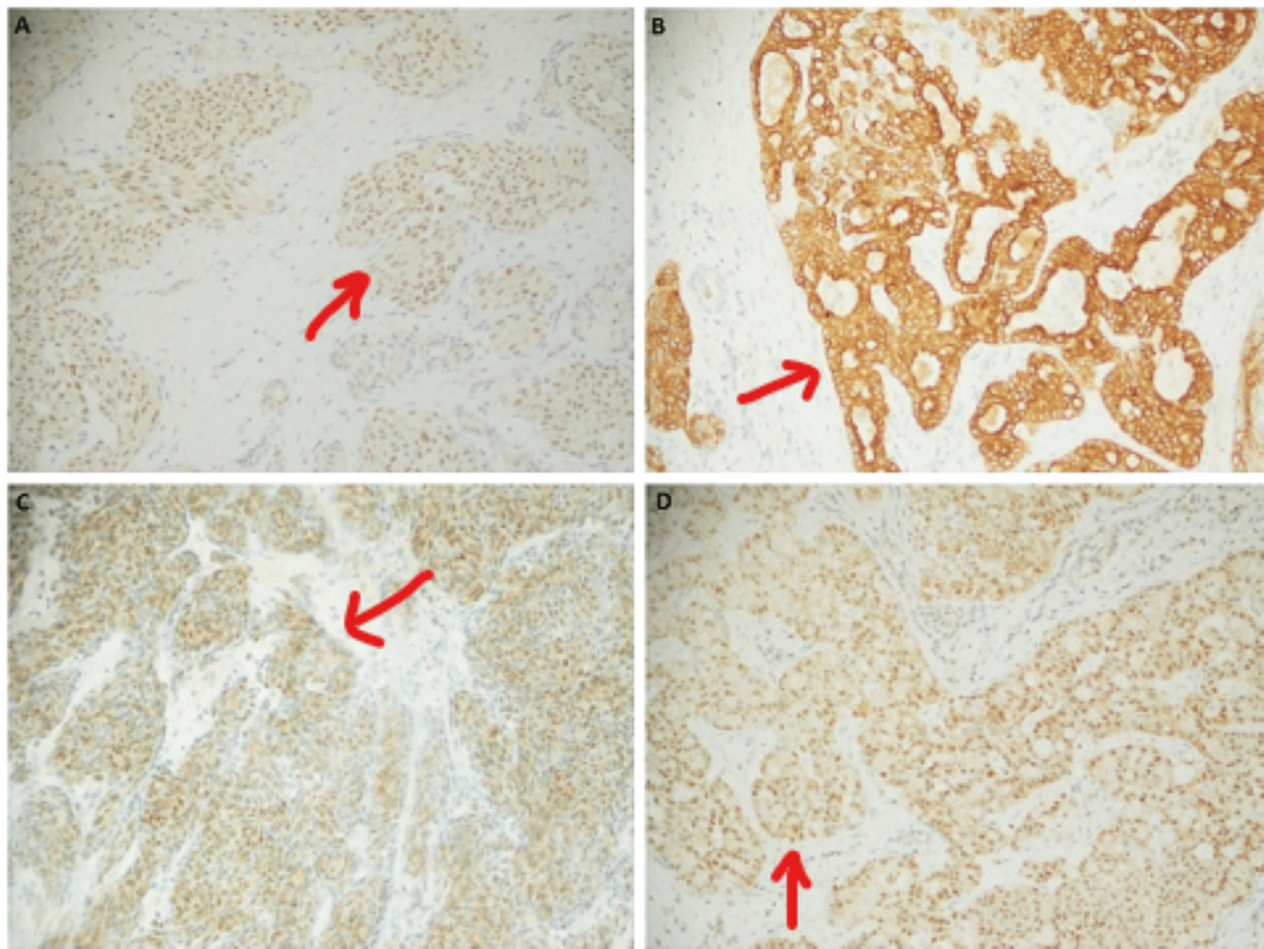
(A): ER: Moderate nuclear staining in 80% of cells; (B): CK-7: Positive in tumor cells; (C): CDX2: Strong nuclear staining, (D): GATA-3: Strong nuclear staining

A 44-year-old male, with no comorbid conditions and no history of cancer, first presented in October 2020 with a history of a left axillary lump for many years. He had his first biopsy in 2011 with benign histopathology and again had an excision biopsy in September 2020, which was consistent with adenocarcinoma with involvement of papillary and reticular dermis with focal involvement of subcutaneous tissue. Estrogen receptor positivity was 80%. Deep margins were free of tumors, and lymphovascular invasion was present (Figures 1-2).

**Figure 3:**
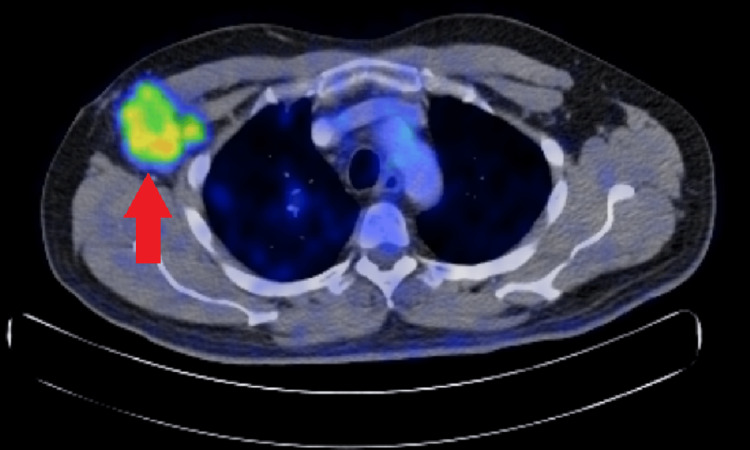
Right axillary nodal mass avid on the PET scan

**Figure 4:**
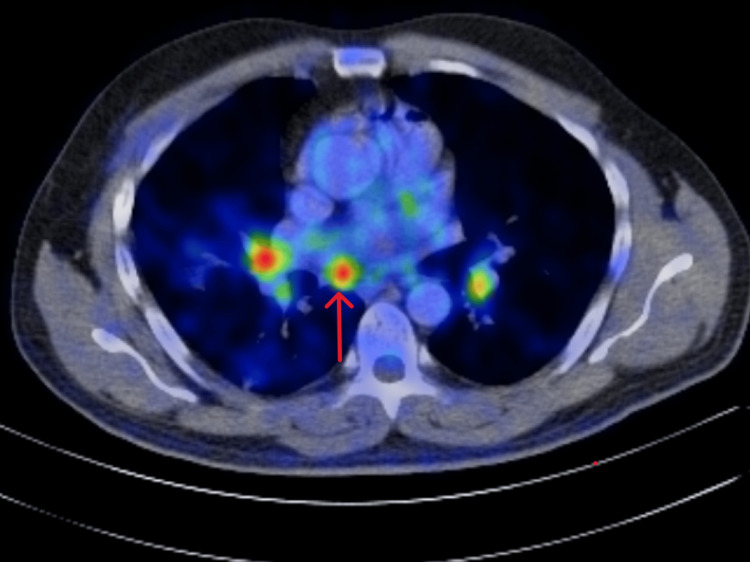
Mediastinal nodes avid on the PET scan

A further staging CT scan suggested the right axillary fossa recurrent soft tissue mass represented apocrine tissue, a primary site of malignancy. This was further investigated via PET-CT, which suggested hypermetabolic right axillary and mediastinal lymphadenopathy with solitary metastatic osseous lesions (Figures 3-4). 

Magnetic resonance imaging of the breast was done, which was inconclusive for any breast lesion.

**Figure 5:**
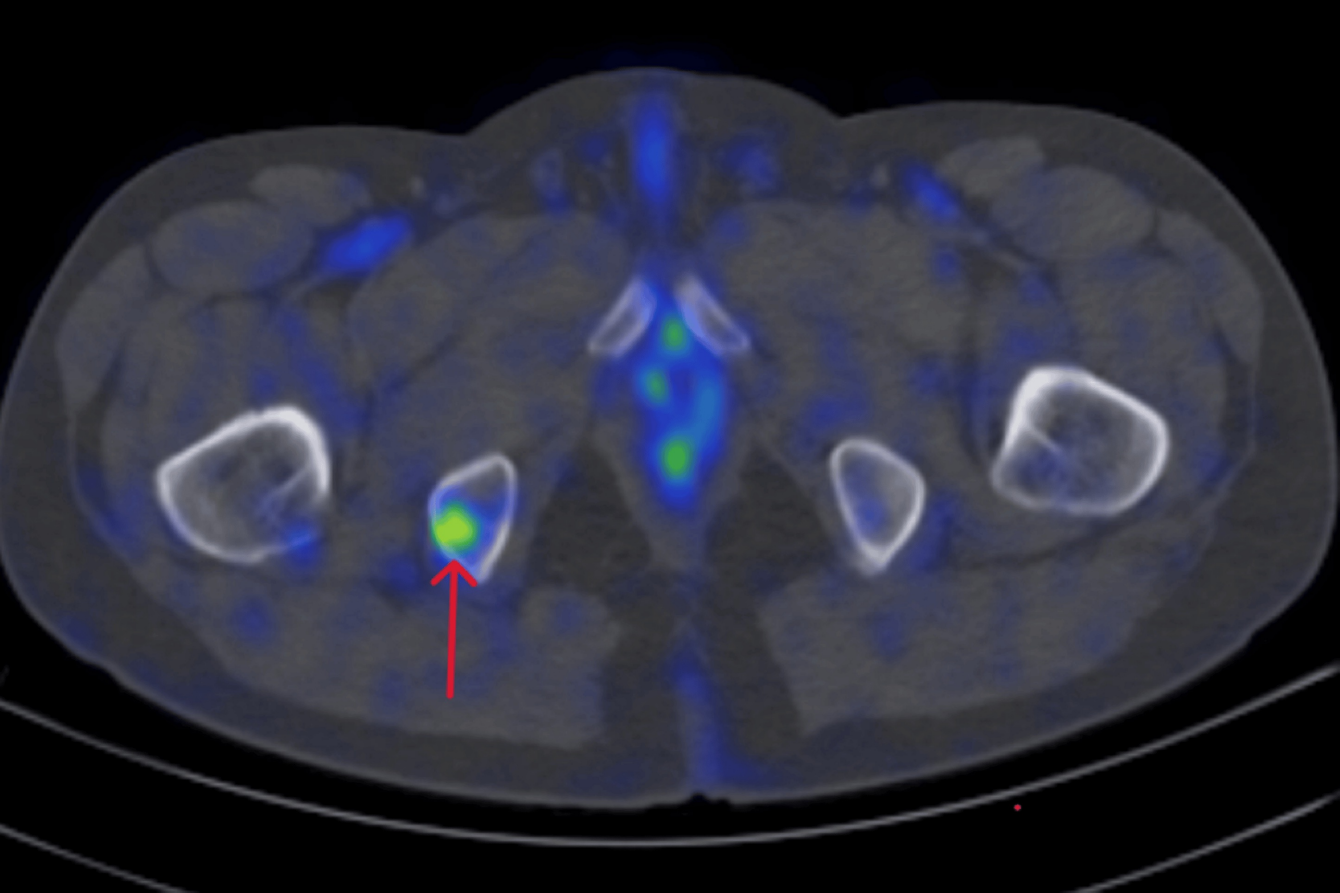
Right ischium lesion avid in the PET scan

After discussion with the patient, he was started on tamoxifen, and a repeat PET CT was done after six months, which suggested progressive disease by virtue of interval increase in the size of the right axillary mass, increase in the metabolic activity of the mediastinal lymph nodes, and new development of hypermetabolic lytic lesion in the right ischium (Figure 5).

After the tumor board discussion, he was started on weekly paclitaxel (80 mg/m²) for 19 cycles; an interim CT after six cycles suggested a stable disease response. Given his stable disease, he was offered axillary node dissection, which was done in November 2021. Histopathology suggested eight out of 15 lymph nodes were positive for metastatic adenocarcinoma with extranodal extension. Skin tissue showed foci of carcinoma in the subcutaneous tissue, with clear margins. Immunohistochemical stains were positive for mucicarmine and focally positive for adipophilin, consistent with mucinous adenocarcinoma of the skin (apocrine glands) in consideration of previous imaging and biopsy results.

After four weeks of surgery, the patient presented with persistent backache. The MRI was consistent with multi-level spinal osseous metastatic deposits and no definitive evidence of cord compression. Further CT scan suggested no local recurrence; however, interval increase in right peri-bronchial thickening along with interlobular septal thickening and patchy ground glass opacities is concerning lymphangitic spread of disease. He was given a single fraction of radiation for pain control. Keeping in view his next-generation sequencing report (Table 1) and disease progression, he was then started on ribociclib/fulvestrant for two months; afterward, his disease progressed with new lung metastasis and cord compression at thoracic and lumbar vertebral levels (T3, L2). The patient was then started on carboplatin/paclitaxel for two cycles. Due to his poor tolerance, chemotherapy was stopped, and the best supportive care was opted for, with the median overall survival exceeding 22 months.

**Table 1:** Next-generation sequencing report of the patient

Biomarker	Result	
Tumor mutation burden	0.72 Muts／Mb	
MSI	MSS	
Content	Therapeutic effect prediction	Result
MLH1 LOF mutation	Positive correlation	Not detected
MSH2 LOF mutation		Not detected
MSH6 LOF mutation		Not detected
PMS2 LOF mutation		Not detected
ATM LOF mutation	Positive correlation	Not detected
BRCA 1 LOF mutation		Not detected
BRCA2 LOF mutation		Not detected
FANCA LOF mutation		Not detected
CHEK1 LOF mutation		Not detected
ATR LOF mutation		Not detected
PALB2 LOF mutation		Not detected
PBRM1 LOF mutation	Positive correlation	Not detected
POLE LOF mutation	Positive correlation	Not detected
POLD1 LOF mutation	Positive correlation	Not detected
PTEN LOF mutation	Negative correlation	Not detected
B2M LOF mutation	Negative correlation	Not detected
JAK 1 LOF mutation	Negative correlation	Not detected
JAK 2 LOF mutation	Negative correlation	Not detected
EGFR GOF mutation	Negative correlation	Not detected
MDM2 increased copy numbers	Negative correlation	Not detected
MDM4 increased copy numbers	Negative correlation	Detected
DNMT3A LOF mutation	Negative correlation	Not detected
STK 11 LOF mutation	Negative correlation	Not detected

## Discussion

Primary apocrine adenocarcinoma is an extremely rare neoplasm that occurs in relatively older people with a median age of 67 years and has a very low incidence of less than one case per 100,000 patients per year [1]. Because of its rarity, prognostic factors are very difficult to establish. The Scarff Bloom-Richardson grading system, already being used in grading and prognostication of breast cancer, was studied in this rare tumor, and it was found that there was a significant statistical relationship between overall survival and the tumor grade [16].

No formal clinical trial is available for treatment purposes; non-metastatic apocrine adenocarcinoma is usually treated with wide local excision with clear margins, and chemotherapy or radiation therapy is reserved only for patients who have high-risk features like large tumor size, positive margins, node-positive disease with extranodal extension, lymphovascular or perineural invasion [2, 6]. As far as locally advanced or metastatic disease is concerned, no consensus guidelines are present in the literature, and it’s difficult to conduct a prospective trial owing to its rarity. As apocrine carcinoma of other sites had many similarities with apocrine carcinoma of the breast, the treatment regimens used to treat this rare tumor are the same as those used to treat breast cancer, including endocrine therapy, which is used in patients with positive estrogen receptor (ER+) [17, 19]. Hollowell et al. reported the median overall survival in patients with localized disease, positive lymph node disease, and metastatic disease of 33, 55, and 14.5 months, respectively. Mostly, tumors recur locally as compared to distant metastasis. Visceral organs, including the brain, bones, and pleura, are the common sites of distant metastasis [20]. 

Our patient was at high risk at presentation as there was a lymphovascular invasion in the histopathology report and was hormone receptor (ER) positive; later, he developed metastasis to the bones. Keeping in mind his metastatic disease, next-generation sequencing testing was done, which helped to confirm the diagnosis and identify mutations for which targeted therapies could be given. Unfortunately, no mutation could be identified in the next-generation sequencing test performed, therefore, targeted therapy could not be offered. However, next-generation sequencing should still be considered to identify the possible mutation and explore the option of targeted therapies. Using this information, we can define the treatment regimens to treat this rare tumor. As it can be noted in the next-generation sequencing report of the patient (Table 1), some mutations have a positive relation with this kind of tumor, so if present, these will help decide and utilize targeted therapies according to the mutations, and this can help in the management of the patient when we have no further treatment options left defined so far for the treatment of apocrine carcinoma. Unfortunately, we could not identify any mutations that had a positive relation in this case, so further treatment options were not available for this patient.

## Conclusions

This case presents a unique diagnostic and therapeutic challenge for a very rare tumor. For primary apocrine adenocarcinomas with aggressive behavior, a multidisciplinary treatment plan may be required. More and more cases need to be reported and followed up for longer durations so that results can be evaluated and treatment regimens can be defined for this rare tumor. Next-generation sequencing testing is required in the future to identify any targetable somatic mutations, which will help in selecting a preferred targeted agent, and this can also be used as a tool to separate this entity from other metastatic diseases. The next-generation sequencing test of the patient didn't reveal any targetable mutations, but it highlighted the mutations that had a positive relation with this tumor. If we find them, we should consider the targeted therapies for those mutations. More and more cases of patients undergoing next-generation sequencing testing and then receiving targeted therapies need to be reported so that outcomes of such patients can be assessed and further guidelines regarding the management of this tumor based on next-generation sequencing testing can be established. 
